# Natural Behaviour Is Not Enough: Farm Animal Welfare Needs Modern Answers to Tinbergen’s Four Questions

**DOI:** 10.3390/ani13060988

**Published:** 2023-03-08

**Authors:** Marian Stamp Dawkins

**Affiliations:** Department of Biology, University of Oxford, Oxford OX1 3SZ, UK; marian.dawkins@biology.ox.ac.uk

**Keywords:** natural behaviour, farm animal welfare, Tinbergen, 4 questions, rewards

## Abstract

**Simple Summary:**

The term ‘natural behaviour’ is widely used by food companies to advertise their animal welfare credentials but the public needs to be made aware that there is no necessary connecton between naturalness and good welfare. Some natural behaviour contributes to good welfare but others, such as being chased and caught by a predator, does not. Used on its own without supporting evidence, ‘natural behaviour’ lacks the most essential criterion for good welfare—whether it matters to the animals themselves. There are now tried and tested methods for establishing what animals value ranging from simple choice tests to what they find rewarding in learning tasks and are prepared to work to obtain. In addition, the growth of animal behaviour studies over the last 60 years has yielded a wealth of new information about what animals respond to, what motivates them, how their behaviour develops during their lifetimes and how the behaviour of modern farm animals differs from that of their wild ancestors. By using all this information as well as just relying on the shaky concept of ‘natural behaviour’, there are thus now opportunities for improving farm animal welfare that are both more evidence-based and more animal-centred.

**Abstract:**

Despite the many scientific objections that have been raise to it, ‘natural behaviour’ is widely used as an indication of good welfare by the food industry. The supposed link between welfare and natural behaviour derives, however, from a now outdated view of animals becoming frustrated if they cannot perform their natural instinctive behaviour. On the 60th anniversary of its publication, Niko Tinbergens’ Four Questions framework is used to show why there is no necessary link between natural behaviour and welfare and why, therefore, reliance on natural behaviour in commercial farming may not result in the claimed improvements in welfare. Used on its own without supporting evidence, ‘natural behaviour’ lacks the most essential criterion for good welfare—*whether it matters to the animals themselves.* There are now a number of well-established methods for demonstrating what animals value, including choice tests and, particularly, what animals will work and pay a cost to obtain. Some of the evidence on what animals value is already available in published papers but some will require collaborative research between scientists and commercial farming to find practical and commercially viable ways of providing animals with what they value.

## 1. Introduction

In 1963, Niko Tinbergen [1] put forward a framework on how to study the behaviour of animals. This framework is often referred to as ‘The Four Whys’ or ‘The Four Questions’ because Tinbergen, while emphasizing the complexity and diversity of animal behaviour, also argued that there were four questions that could always be asked about any behaviour — about how it develops (ontogeny), about how it helps an animal to survive and reproduce (adaptation), about how it has changed over evolutionary time (phylogeny) and about the neural, hormonal and other mechanisms underlying the behaviour (causation). These four questions have also been immensely influential in the development of a scientific approach to animal welfare [2,3,4] and have stood the test of time precisely because they are both specific enough to be clear that different answers are required to the different questions and general enough to accommodate the many advances in genetics, neuroscience, behavioural ecology, animal welfare science and evolutionary biology that have occurred since 1963. 

However, there is one application of animal behaviour where the progress that has been made since Tinbergen first posed the Four Questions has been largely overlooked, or perhaps just not updated. This is the widespread reliance on ‘natural behaviour’, as a way of assessing animal welfare. ‘Natural behaviour’ is commonly defined as behaviour shown by animals living where their ancestors evolved or at least in man-made environments that allow them similar freedom of movement [5,6]. The welfare of more confined members of the same species — such as those living in zoos, farms or in peoples’ homes — is then judged by the extent to which they, too, are able to show this natural behaviour.

Despite the many scientific criticisms that have been levelled at the idea of linking natural behaviour to animal welfare e.g., [5,6,7,8,9,10,11,12,13,14,15], natural behaviour is now widely used as a welfare metric outside science, particularly by people in the food industry responsible for company animal welfare policies. The ability to perform natural behaviour is made explicit in the sustainability and ethical goals of many food producers, food retailers and outlets across the world. For example, a major international food outlet gives as one of its animal welfare priorities “Providing enrichments that support natural behavior” and in applying this specifically to chickens states: “We are working with supply chain partners to ensure housing environments that promote natural behaviors such as pecking, perching and dust-bathing.” [16]. 

Given the widespread use of natural behaviour as a welfare metric by the food industry and others, there is clearly a need to explain to those with the power and money to make changes to the way farm animals are managed exactly why over-reliance on natural behaviour may not improve welfare in practice as much as they hope and that there are more effective evidence-based ways of improving the lives of farm animals [17]. 

The aim of this paper is therefore to show how animal welfare on commercial farms could be improved by relying less on uncritical use of natural behaviour as a welfare metric and by giving more weight to evidence of what the animals themselves actually value. By asking Tinbergen’s four classic questions but answering them in the light of more recent knowledge of animal behaviour, it becomes clear why we need to look beyond natural behaviour towards more animal-centred ways of assessing welfare.

## 2. Natural Behaviour and Welfare

Natural behaviour began to feature prominently in public discussions of farm animal welfare following the publication in 1979 of the Five Freedoms [18], five simple statements about what is necessary for good animal welfare. The first three of the Five Freedoms address the conditions needed for physical health — freedom from hunger, freedom from physical discomfort and freedom from injury and disease, while the last two refer to mental health — freedom from stress and, significantly, the freedom “to perform most normal patterns of behaviour”. (‘Normal’ rather than ‘natural’ was used to allow for the fact that domesticated farm animals may behave differently from their wild ancestors but still have the same need for freedom of movement). 

W.H. Thorpe [19] expressed a common ethological view at the time in this way: “A very large part of animal behaviour is basically determined by instinctive or innate abilities, proclivities and dispositions. Suppression of these instinctive appetites can give rise to evidence of prolonged and intense emotional disturbances which, whether or not they are painful to the animal, are most distressing to see.” (p. 73). Referring specifically to farm animals such as chickens and pigs, Thorpe went on: “ Whilst accepting the need for much restriction, we must draw the line at conditions which completely suppress all or nearly all the natural, instinctive urges and behaviour patterns characteristic of actions appropriate to the high degree of social organization as found in the ancestral wild species and which have been little, if at all, bred out in the process of domestication.” (p. 79). 

Thorpe thus brought together the idea of animals having innate instinctive urges or needs to perform their various natural behaviour pattens put forward by Konrad Lorenz [20,21] with the idea that if these urges were suppressed, this resulted in emotional disturbance. He then went further and concluded: “What is required is to examine the incidence of those expressive movements which are known to be associated with damaging situations in order to assess whether animals brought up with a certain degree of deprivation “suffer” from deprivation and stress in adulthood.” (p. 79). By using the word “suffer” Thorpe broke the prevailing scientific taboo on discussing what animals might consciously feel and made the connection between natural behaviour, instinct and animal welfare that has persisted to this day. 

Being able to perform natural behaviour has also become, for some, what good welfare means, an expression of animal’s essence or *telos* and the way it was evolved to be in its natural environment [15,22,23]. This view is also reflected in surveys of public opinion, which consistently sees natural behaviour as an essential part of animal welfare [24,25,26,27,28]. 

Natural behaviour can thus easily appear to be the ideal welfare metric. It is based on the animals’ own behaviour, it can be measured and even quantified and above all it has public opinion on its side. All this makes it easy for the food industry to use the emotional impact of the word ‘natural’ in promoting their products and to overlook the very real problems that exist with linking natural behaviour to welfare. These are so substantial that natural behaviour, if taken on its own, may not improve animal welfare at all. To see why, we can use Tinbergen’s four questions. 

## 3. Tinbergen’s Proximate Questions—Development and Causation

The idea articulated by Thorpe [19] that most animal behaviour is driven by instinctive urges has now been largely discarded. It was based on a once influential model of animal behaviour put forward by Konrad Lorenz [20,21] in which he argued that animals had motivational energy or drive associated with each of their behaviours which built up inside them the longer they had gone without doing that behaviour. When the behaviour was actually performed, the motivational energy would be discharged and the animal would no longer want to do the behaviour. If the animal were prevented from showing the behaviour, the level of motivational energy would rise and ‘dam up’ until eventually it would spill over and result in inappropriate and damaging behaviour. ‘Damming up’ was then used as evidence by Lorenz and others that animals had behavioural needs that had to be met to ensure good welfare [21,29,30]. 

The time course of some behaviour does indeed suggest that something might be accumulating inside a deprived animal, resulting in a greatly increased tendency to perform that behaviour when the opportunity eventually arises [31]. This ‘rebound’ effect [32,33] is shown by people who make up for lost sleep by sleeping more when eventually able to rest, by chickens that make up for deprivation of dust-bathing [31] and cows that make up for deprivation of lying [34,35] by doing more when they can. 

Even 60 years ago, however, Lorenz’s model with its implication of damming up of instinctive urges had been heavily criticized [36]. Quite apart from its reliance on unspecified motivational energy, it simply failed to explain much real behaviour [36,37,38,39], leading most scientists to discard it. However, its influence lived on. Even as the model itself was consigned to history as not explaining the behaviour of most real animals [40], the idea that it gave rise to—that animals have instinctive motivational drives to perform all their natural behaviour—survived and still influences the way many people see animal behaviour to this day. 

Lorenz [20] also argued that each species had its own repertoire of instinctive behaviours consisting of relatively fixed units or patterns of behaviour, such as pecking or scratching, which he called Fixed Action Patterns. He saw these Fixed Action Patterns as the basic building blocks of behaviour, performed more or less in the same way each time and then linked together to form sequences characteristic of a given species. 

Some behaviour does indeed seem to have a fixed, instinctive element such as nest-building in sows [41], which can be passed without learning from generation to generation but most behaviour is also flexible and changes according to the experience of the individual. Behaviour is controlled by a variety of different mechanisms that develop with different mixtures of genetic, environmental and social influences [42]. It is thus no longer possible to treat natural behaviour as a single category, with all behaviour having the same welfare consequences if it is prevented or not performed [40,42,43,44,45,46]. Each behaviour needs a separate understanding of its causation, development and what happens if an animal is unable to perform it. The strength of the link to welfare will also vary.

Natural behaviour can even be a sign of poor welfare. Behaviours such as being caught by a predator are entirely natural in the sense that they occur in nature, but they do not necessarily improve welfare as judged by either physical health or what the animal themselves want [9,11,13,15,46]. Natural behaviours such as hiding from a predator, scratching or constantly searching for food occur not when the animal’s welfare is good but when it is poor, such as being in a state of continuous fear, physical discomfort or with a deficit of a vital nutrient. Indeed, much natural behaviour of wild animals can be seen as attempts to ward off the constant threats of death by predation, disease, malnutrition, attacks by other animals, parasites and adverse weather that wild animals face for much of their lives [13,47]. 

Natural behaviour, taken on its own, is thus a very unreliable indicator of the welfare state of the animal. Without additional evidence of what happens if a particular behaviour does not occur, it is impossible to establish a convincing link to welfare because natural behaviour on its own lacks the most essential indication of welfare—whether performance or non-performance of a behaviour actually *matters to the animal* [5,48]. 

A major advance in the study of behaviour over the last sixty years has been the development of ways of finding out what does matter to animals. It is now possible to effectively ‘ask’ animals what they want and Tinbergen’s questions about development and causation can now include answers about the effects of performing or not performing natural behaviours. It is this previously missing information about what matters to the animals themselves that now needs to be added to the uncritical use of natural behaviour as a welfare metric. 

## 4. What Animals Value 

As Lorenz and Thorpe both recognized, the ability to learn makes animals much more flexible than if they had to rely solely on a fixed set of instinctive behaviour patterns [49,50]. For example, an animal may eat foods that come in a variety of shapes and sizes, are found in a variety of different places and require a variety of techniques to obtain the nutritional content from them. By having an innate liking for, say, a sweet taste and combining this with an ability to learn many new behaviours to obtain that sweet taste, the animal can achieve far more flexibility and adaptability to a changing unpredictable world than if its feeding behaviour involved always doing the same ‘natural’ behaviour over and over again. It could exploit new foods never encountered by its ancestors and quickly learn to take advantage of new ways of obtaining nutrition, even though these might be quite ‘unnatural’ and never seen by any wild ancestor. 

Far from being fixed and instinctive, most animal behaviour is a subtle and constructive interplay between genes and environment [42]. Genes specify what an animal finds rewarding (such as a sweet taste) or punishing (such as pain) and so guide it towards what is beneficial and away from what is harmful. But the genes do not usually specify exactly what behaviour has to be doneto achieve these ends. The animal has to learn from its own experience how to reach rewards or avoid punishments [50,51]. By discovering what those rewards and punishments are, and how hard animals will work to obtain or avoid them, we have an objective way of knowing what they value.

Rewards and punishments are at the heart of current theories of both human and animal emotion, [50,51,52,53,54,55]. Indeed, emotions are defined as states elicited by the presentation, termination or omission of rewards and punishers. Thus, a positive emotion of pleasure might be elicited by receiving a reward such as food when hungry, social approval or warmth when cold while a negative emotion such as fear might be elicited by an impending punishment such as a rapidly approaching predator. Some emotions such as frustration are elicited by not receiving an expected reward or having it taken away, while yet others, such as relief come about when an expected punishment is removed or avoided [50]. This way of classifying emotions by whether they are positive or negative (sometimes called their ‘valence’) is readily applicable to animal welfare [54] since good welfare can then be seen as animals having predominantly positive emotions [54,56,57,58]. Adding a second dimension of arousal or strength of emotion makes the approach even more relevant since a distinction can then been made between mild or transitory negative emotions, such as a mild itch that is of minor concern and those that are both negative and strongly aroused and so deserve the label ‘suffering’. 

The idea of using what animals value as judged by the rewards they will work to obtain and the punishers they will work to avoid is now well established as an important part of an animal-centred approach to animal welfare [5,17,29,57,59,60,61], and it has changed how the link between natural behaviour and welfare is now perceived. What animals have been evolved to do is not so much to ‘perform a behaviour’ but to be rewarded or punished by circumstances that are the usual result of such behaviour [50]. This means that we should not be assessing welfare just by whether animals are showing natural behaviour but by whether they have what they value, as shown by what they will work for. Not all natural behaviour turns out to have the same value to an animal [5,6].

To show that an animal values something means showing that it will ‘work’ for a reward—that is, it will learn to perform some arbitrary action to obtain it. For example, laying hens will learn to peck a key to gain access to straw [62] and pigs, calves and shorn sheep will learn to operate switches for the reward of heat [63,64,65]. Heifers can learn to operate a panel with their noses to give themselves 15 min of being able to lie down [66] and calves push a panel with their heads for the reward of a few minutes’ social contact with another familiar calf [67]. 

Making animals work for their rewards, however, is often impractical as it involves equipment that is not available on farms. Even more seriously, it may be logistically difficult with some rewards to present them repeatedly. With food as the reward, small amounts can be delivered on each trial so that the animal has to repeat the response if it wants more of the reward but with other rewards such as lying down or dustbathing this is more difficult. The animal has somehow to be removed from what it has chosen the first time so that it can make another response and this may mean being handled, pushed or shuttled away from what it has just chosen to see whether it still wants it the next time. For example, to show that young cows want to lie down enough to learn to operate a switch for the reward of lying down for 15 min, Jensen et al. [66] had to make them stand up again after 15 min so they could make another response. Apart from the logistical problems involved in devising the appropriate equipment, being made to stand up every 15 min could itself have made the opportunity to lie down less rewarding for the cow. Practical considerations thus mean that what animals value has often to be measured in other ways. These include:

### 4.1. Spatial Distribution

The ways animals position themselves within their environment, or in relation to each other can be particularly useful for identifying what animals want when formal tests are simply not feasible, such as in investigating whether animals want more space or find high stocking densities aversive. Because they do not involve disturbing the animals in any way, such methods can be very sensitive and reveal subtleties in what animals want that other more intrusive methods can miss [68]. When they are very young, for example, broiler chickens choose to cluster together and want to be together [69] but when they are older, they tend to space themselves out and find it aversive to be too close to other birds [70]. 

### 4.2. Time Spent

Looking at spatial distribution over time gives an even clearer idea of what animals want, particularly if they move around and so make repeated choices about where they want to be. Measuring the amount of time animals spend on or near resources such as perches, shelters or scratching areas is a relatively easy, non-disruptive way of seeing which ones they value most by where they spend most of their time. It can also help to rule out ones that they do not value. If something is claimed as an ‘enrichment’ and the animals take no notice or avoid it altogether, then it can hardly be claimed that they value it. Zoos often use this method to find out whether animals find the presence of visitors aversive or attractive by providing refuges where the animals can hide from visitors and then seeing whether the animal choose to spend time close to visitors or keep away from them [71,72,73]. It is also possible to establish what physical features of their enclosures animals like or dislike by where they spend their time. In an agricultural context, both free-range broiler chickens and free-range laying hens spend a disproportionate amount of their time either under or near trees if this option is available to them [74,75] and cattle choose to spend time lying down outside, particularly at night [76]. 

### 4.3. Approach/Avoidance

A somewhat more structured and controlled way of assessing what animals value is to measure their speed of approaching or moving away from something. If this speed increases (or decreases) the more experience they have of it, this indicates whether they have found the experience rewarding (or punishing). Hanmer et al. [77] trained rats to run down a corridor to a goal box where they were allowed 5 s interaction with a training object. Once the rats were reliably running to the goal box, the training object was removed and replaced by different potential ‘enrichment objects’ such as a plastic ball or a cardboard tube and the rats were given three more trials with each new object. Over these three trials, rats ran at different speeds depending on what object they were running towards in the goal box. They ran fastest towards objects that they had previously shown they preferred in a two-way choice test. Running speed thus gave a continuously varying indication of how much value they put on interacting with the different objects.

Rushen [78] used a decrease in speed of approach to show that sheep find the controversial practice of electro-immobilisation (in which the sheep are stopped from struggling while being sheared by having electric current passed through them) particularly aversive. Sheep were put at one end of a corridor which they naturally ran down to escape from people at the start end. At the other end of the corridor, some of the running sheep were caught and physically restrained by a person (as would happen in normal shearing), others were caught and then electrically immobilised, (which makes things much easier for the shearer) and others were not restrained at all. Once the sheep had had experience of what was going to happen to them at the end of the corridor, they were individually put back at the start of the corridor and their running speed was measured. Sheep that had experienced the full restraint with electro-immobilization moved more and more slowly as the trials progressed and eventually became reluctant to approach the end of the runway at all, suggesting that this procedure was something they wanted to avoid. Sheep that had simply been restrained without electro-immobilisation, moved faster but the sheep that were never caught at the end of the runway ran fastest of all, suggesting that being restrained by a human is something sheep want to avoid if they can. However, they want to avoid electrical immobilisation more than anything.

### 4.4. Choice and Preference Tests

These can also be thought of as approach or avoidance tests, but the animal is offered two or more options at the same time and so is indicating the relative value it puts on the various options. Although choice tests can be conducted on a small scale, such as offering an animal a choice of two or more types of, food, flooring or types of bedding material, they can also be used on a grander scale to provide answers to questions such as whether farm animals should be kept outside and allowed to range freely. For example, human consumers are often critical of the increasing practice of keeping dairy cows indoors and believe that cows should be able to go outside and eat grass [79]. It is therefore of considerable public interest to know whether the cows themselves value going outside. Charlton et al. [80] gave dairy cows a choice between indoor housing where they could obtain high energy food and going out to pasture where they could graze freely. Twice a day after milking, they took individual cows to a choice point equidistant (48 m) from indoor housing and pasture and let them choose where to go. Between milkings, the cows could move between the two areas so that they had ample experience of both. When taken to the choice point, the cows were more likely to choose the pasture than the indoor stalls (66.2% vs. 33.8%) but overall spent more time indoors, although the behaviour was highly dependent of the weather, the time of year and the time of day [81,82]. Choice tests have also shown that pigs and chickens are very sensitive to the smell of ammonia and choose to avoid it even in concentrations as low as 10 ppm [83,84].

### 4.5. Conditioned Place Preference/Aversion 

Sometimes it is quite impossible to use any of the above methods to discover what an animal does or does not value because all these methods require an animal to have repeated experiences so that it can give an ‘informed’ opinion of what is happening to it. Where something happens only once—such as being caught or having a one-off medical procedure—it is not possible to see whether the experience results in a change of behaviour, however negative or positive the experience might have been, because there is no next time.

Under these circumstances Conditioned Place Preference (CPP) or Avoidance (CPA) can be very useful methods as they involve training animals, with a single trial, to associate one place with a particular experience such as food or a loud noise and a second place with something completely different, usually where nothing happens [85]. The two places are made very obviously different such as being painted with different colours or patterns to make it as easy as possible for the animal to associate each place with what happened to it there. The animal’s preference for being in one place or another is measured both before and after the experience being investigated. If, after the single experience there is a shift in where the animal chooses to spend its time so that it starts to spend more time where it obtained a reward, this suggests that it liked the experience and is trying to repeat it [86]. Conversely, if it now avoids the place where the stimulus appeared and starts to prefer the place where it did not experience it, then this suggests that it found the stimulus aversive and wanted to get away from it. The advantage of this method is that it can work with just one experience, provided that the animal is clever enough to make the association.

### 4.6. Instrumental Conditioning

Even though methods described in Section 4.1, Section 4.2, Section 4.3, Section 4.4 and Section 4.5 provide important information about animal preferences with relatively little or no special equipment and often with minimal interference with the animals themselves, demonstrating what they find rewarding or punishing using operant conditioning is still the best test of what they value having (or being able to avoid). This is because approach/avoidance and expressions of preference can all be hard-wired, innate responses (such as reflexes, taxes or kineses), done automatically and unthinkingly. Bacteria can exhibit ‘preferences’ by moving up a chemical gradient and even plants can appear to make choices by growing towards light or away from the pull of gravity.

Goal-directed, action-outcome ‘working’ for rewards, however, suggest an altogether more complex mechanism, involving different brain pathways [48,85]. The easiest way to distinguish a mechanism based on rewards and punishments from simpler mechanisms (such as always turning left to find food), is to make a reward contingent on the animal making a completely arbitrary response, such as pressing down on a lever or turning in a circle and then seeing whether the animal will learn to do the exact opposite and push the lever upwards or circling in the other direction when the reward contingencies change [49,50,87,88]. If the animal can continuously update its behaviour and do whatever it takes to get at the reward, then clearly there is no fixed relationship between the stimulus (e.g., the sight of food) and the response and we have correctly identified that the animal’s goal is obtaining the reward.

## 5. Rewards, Punishments and Welfare

Even if an animal will work for a reward, however, this still does not complete the link to welfare. To do this, we need to know whether obtaining what is being approached, preferred or worked for actually matters to the animal. In other words, we need to know just what an animal wants but how much it wants it in comparison to something we know is important to it, most obviously food [89,90]. One way of doing this is to make it more difficult for an animal to access a reward and then to see if it wants that reward so much that it will overcome the difficulties and effectively pay a higher price for every reward. If it is prepared to pay an ever -higher price—and particularly if it is prepared to pay the same price as it would for food—then we have an objective measure of what it values.

The way a price is paid can be varied to suit the animal and the circumstances. To demonstrate what they value, animals have been willing to squeeze through a narrow gap [91,92], walk down a long corridor [93], push a weighted door [80,94,95,96], walk through a water bath [97,98], run over an electric grid [99], be blasted with a jet of air [100] or simply repeatedly perform an operant response such as pecking a key not just once but many times for each reward [101]. The maximum price an animal is prepared to pay in the face of these increasing difficulties before it gives up and refuses to pay the price can then be used as a measure of how much it values that reward [101,102,103,104]. If the animal will pay as much for access to bedding material as it will pay for food, it is showing (at least at that moment) that it values bedding as much as food. For example, dairy cows are willing pay a considerable price for access to automated rotating brushes, which they then rub against different parts of their bodies [105]. The cows quickly learn to operate switches to make the brushes rotate and they will push open heavy gates to get access to the automated brushes, paying as high a price (in weights) as they will to get at food when food deprived for 1.5 h [106]. 

## 6. The Ultimate Questions—Survival Value (Adaptation) and Phylogeny

Natural behaviour is, as we have seen, behaviour shown by wild or free-range members of a species that evolved as adaptations to the ancestral environment. Tinbergen [1] argued that questions about adaption could only be sensibly answered by studying animals in their natural habitat—where doing or not doing the behaviour could be demonstrated to make the difference between life and death. It might therefore seem irrelevant to ask this question about domesticated or captive animals that may now be in environments that are very different from those in which their ancestors evolved and are largely protected from the hazards their wild ancestors faced. They have been selectively bred by humans so that many now look and behave very differently from any wild animal. However, there are two important reasons why understanding the adaptive significance of behaviour in wild species is important to welfare.

The first reason is that behaviour that has been critical to survival in an ancestor is a prime candidate for being valued by its descendants, so might fruitfully be one of the first things to be investigated in any research project. For example, the junglefowl ancestors of our domestic chickens roost in trees at night to avoid predation, and this highlights the possible importance of roosting to modern breeds. The fact that wild junglefowl roost in trees does not prove that all modern chickens want to roost, but it provides a very plausible hypothesis that this is might be a behaviour they value. This hypothesis can then be tested. Some behaviour that is necessary to survival in wild species will turn out to be of reward value to their domesticated descendants, others will not, but understanding the adaptive significance of behaviour contribution is a useful way of deciding which hypothesis to test first.

The second reason why the adaptive significance of behaviour in wild ancestors is important for farm animal welfare is that this can often help us to understand why their descendants, living in a completely different, man-made environment can sometime behave in ways that are positively harmful to themselves. Some people see the fact that animals do not always choose what is good for their health in the long run as a serious objection to using what animals value as a welfare metric. Animals may eat things that are poisonous to them, for example, or eat too much and become life-threateningly obese. This is because they are still valuing the rewards that their ancestors valued but in their current, domesticated environment, these rewards no longer align with what is best for their health. Wild carnivores, such as the ancestors of our dogs, often have to chase many prey unsuccessfully before finally securing anything to eat [107]. Even when they have brought down prey, they may have to face competition for it from conspecific or other scavenging species so a tendency to eat quickly whenever food becomes available is key to survival. Transfer this ‘wolfing’ of food to a domestic environment and it can easily lead to over-eating. The lesson here is to use what animals value in the here and now as an important part of their welfare, but to realise that it is not the only measure of welfare that is important. It must be considered alongside physical health as animals, like humans, do not always choose what is best for them in the long run [8]. Animal welfare involves a balance between what is best for long-term health and what is valued in the moment—just as it does with us humans. 

Here we can take a lesson from Tinbergen’s careful separation of four different questions about animal behaviour. He was very clear that questions about adaptation needed quite different answers from those about causation and that confusing the two sorts of question could lead to error. His own work on egg-shell removal in black-headed gulls showed that the adaptive significance of the birds removing empty egg shells from the nest soon after a chick had hatched was protection against predators [108] but he was quite clear that this finding showed nothing in itself about the causal mechanisms that made the gulls remove egg shells. Although the behaviour was an anti-predator adaptation, it was not stimulated by the arrival of a predator as the action had to be taken pre-emptively. The stimuli eliciting the behaviour and the adaptive effect were quite separate, although in the gulls’ wild environment, the two worked harmoniously together. This is the way natural selection works—favouring a mechanism that increases fitness in a given environment. Change the environment and the harmony can be broken and the animal behaves maladaptively.

## 7. Phylogeny

Tinbergen’s final question—about the phylogeny of behaviour—is perhaps the least researched question in the context of natural behaviour and welfare but it deserves more attention than it is generally given. Tinbergen himself was concerned with how behaviour changed over time at the species level—how, for example, a courtship display in a descendent species may be an exaggerated version of a display in its an ancestral species [109]. Most of our domestic and farm animals are still the same species as their wild ancestors (junglefowl and chickens can still interbreed as can pigs and wild boar) but nevertheless, there have been major changes in body shape, behaviour and other traits as shown by the many different breeds of chickens, cows, horses and other domestic animals that have been produced by artificial selection [110]. This constitutes another problem with using the natural behaviour of wild animals as the yardstick for welfare, namely, that in the course of time, breeding by humans to suit human needs has often changed the behaviour and physiology of our farm animals to such an extent that their needs and values are now entirely different from those of their wild ancestors. The farm animals we have now are the complex result of recent artificial selection combined with an ancient legacy from past natural selection. They are neither completely ancient nor completely new. For this reason, we need research to understand their needs as they are now—genetically changed, reared in a domestic environment—the result of both natural and artificial selection.

Artificial selection by humans has much in common with natural selection in the wild, but there are also important differences [111]. One of the most important of these is that humans often select for single traits and then find that this leads to unforeseen problems [112]. For example, selection for young high growth rate in broiler chickens has led to skeletal and other problems including obesity in the adult birds that are used for breeding so that they then have to be feed-restricted, which is itself another welfare issue [113]. Natural selection, on the other hand, is multi-trait. Every change, such as an increase in growth rate has to result in a net overall improvement in survival and reproduction so that a bird that grew faster but could not walk and or reproduce would be selected against. In nature, only faster growing individuals that could walk and produce offspring would contribute genetically to the next generation, whereas in domesticated environments, individuals that would not survive in the wild can be not only be kept alive but bred from.

Humans have selectively bred animals with traits that humans favour such as fast growth rate, milk yield or docility, usually with little heed or knowledge of other traits that may have been selected either as a by-product or as the underlying mechanism. For example, selecting chickens and pigs for fast growth rate has involved changes to their anatomy and their metabolism, affecting how much they eat, how hungry they become, how well they can deal with heat stress and many other aspects of their bodies and behaviour that we are only just beginning to understand [114]. Artificial selection has affected gene expression, brain structure and hormonal balance and countless other traits [115,116]. Understanding what we have done—the phylogeny of domestication—is an important contribution to welfare [110].

## 8. Conclusions

The current emphasis by many in the food industry on natural behaviour as a key requirement for good welfare does not ensure that farm animal welfare is, in practice, improved. Some natural behaviour is associated with good welfare but some is not. The growth of animal behaviour studies over the last sixty years has shown that different natural behaviours have different causes, different developmental pathways, different health outcomes and consequently different implications for welfare. In particular, natural behaviour, if taken on its own, lacks an essential piece of evidence about welfare—namely whether it is valued by the animals themselves. It is this information that is needed to justify claims for improved welfare.

There are now many different methods available for discovering what animals value, ranging from simple observations of what they approach or avoid to showing that they will work to obtain what they want. Some of the relevant information is already available in the scientific literature, while some will need further research to answer specific questions about the role of individual behaviours in each species. Differences in welfare requirements between breeds, sexes and individuals of different ages within one species will also need to be taken into account as well. Collaboration between academia and commercial companies will be essential as even when it is established what animals value, it will still be necessary to find ways of providing it for them that work in practice on a large scale. Changes that are difficult for busy farm staff to handle or clean, or that make an operation uncommercial will not, in the end, improve the welfare of animals.

Tinbergen’s Four Questions, published 60 years ago, still provides the best framework for studying animal behaviour, giving a constant reminder of the complexity and diversity of behaviour and of the many different factors we need to know about before we can with confidence claim to have understood the consequences of an animal showing, or failing to show, a particular behaviour. The framework shows us the importance of understanding what the natural behaviour of a species is, but also provides a warning that much it may have changed through selective breeding and that what is ‘natural’ may or may not be valued by our present-day domestic farm animals.

## Data Availability

Not applicable.
